# Is public transport a risk factor for acute respiratory infection?

**DOI:** 10.1186/1471-2334-11-16

**Published:** 2011-01-14

**Authors:** Joy Troko, Puja Myles, Jack Gibson, Ahmed Hashim, Joanne Enstone, Susan Kingdon, Christopher Packham, Shahid Amin, Andrew Hayward, Jonathan Nguyen Van-Tam

**Affiliations:** 1 Division of Epidemiology & Public Health, University of Nottingham, Clinical Sciences Building, City Hospital, Nottingham, NG5 1PB, UK; 2 St Luke's Surgery, Radford Health Centre, Ilkeston Road, Nottingham, NG7 3GW, UK; 3 Public Health Directorate, Nottingham City Primary Care Trust, 1 Standard Court, Park Row, Nottingham, Nottinghamshire, NG1 6GN, UK; 4 Department of Infection and Population Health, University College London, Royal Free Campus, Rowland Hill St, London, NW3 5PQ, UK

## Abstract

**Background:**

The relationship between public transport use and acquisition of acute respiratory infection (ARI) is not well understood but potentially important during epidemics and pandemics.

**Methods:**

A case-control study performed during the 2008/09 influenza season. Cases (n = 72) consulted a General Practitioner with ARI, and controls with another non-respiratory acute condition (n = 66). Data were obtained on bus or tram usage in the five days preceding illness onset (cases) or the five days before consultation (controls) alongside demographic details. Multiple logistic regression modelling was used to investigate the association between bus or tram use and ARI, adjusting for potential confounders.

**Results:**

Recent bus or tram use within five days of symptom onset was associated with an almost six-fold increased risk of consulting for ARI (adjusted OR = 5.94 95% CI 1.33-26.5). The risk of ARI appeared to be modified according to the degree of habitual bus and tram use, but this was not statistically significant (1-3 times/week: adjusted OR = 0.54 (95% CI 0.15-1.95; >3 times/week: 0.37 (95% CI 0.13-1.06).

**Conclusions:**

We found a statistically significant association between ARI and bus or tram use in the five days before symptom onset. The risk appeared greatest among occasional bus or tram users, but this trend was not statistically significant. However, these data are plausible in relation to the greater likelihood of developing protective antibodies to common respiratory viruses if repeatedly exposed. The findings have differing implications for the control of seasonal acute respiratory infections and for pandemic influenza.

## Background

The current UK National Framework for Pandemic Influenza states that during a pandemic, domestic travel should continue to operate normally but users should adopt good hygiene measures, stagger journeys where possible to reduce overcrowding; and stay at home altogether if symptomatic with pandemic influenza [1]. This advice reflects the need to maintain, as far as possible, business continuity and near normal functioning of society, but acknowledges that some data exist about the transmission of influenza on board public transport, notably commercial airliners [2].

Until very recently, there were no data that directly supported or refuted an association between the use of public ground transportation and the risk of acute respiratory infection (ARI). The risk posed by large numbers of transient casual human contacts has not been adequately defined. However, a recently published cohort study of hospital healthcare workers suggested no association between serologically confirmed seasonal influenza illness and regular public transport use; however recent use just before the onset of respiratory illness was not evaluated [3]. A retrospective investigation following an outbreak of pandemic influenza A/H1N1 2009 amongst school children, noted no forward transmission from the index case to contacts despite probable exposure on a school bus for up to 60 minutes [4]. The current uncertainty makes the formulation of pandemic transport policies difficult.

## Methods

### Study design and population

A case control methodology was employed. We recruited from patients registered at an inner city General Practitioner (GP) surgery in Nottingham, who consulted a doctor between December 2nd 2008 (week 49) and January 15th 2009 (week 02), a period which coincided with known community influenza activity, and which peaked in week 51 [5]. The study involved self-completion of a questionnaire eliciting acute symptoms; socio-demographic characteristics; health status including co-morbidities; and transport habits generally, and specifically in relation to the period immediately preceding the onset of illness. Patients of all ages were eligible for inclusion, provided they were registered with the recruiting surgery, and had presented to the surgery with an acute condition with a date of onset during the designated study period. For patients under the age of 14, the questionnaire was completed on their behalf by a parent or guardian. Patients presenting with chronic or recurrent conditions, visiting the doctor for repeat prescriptions, advice, or health maintenance or monitoring (general check-ups and screening activities) were excluded from the study. Questionnaires were either completed by the patient at the surgery (assisted if necessary by the resident researcher, JT), or at home, then posted back in a pre-paid envelope.

### Case definition and exposure ascertainment

Cases of ARI were identified, based on clinical diagnoses made by the three participating GPs. A case-specific questionnaire also identified any use of buses and trams in the five-day period before symptom onset, representing a maximum putative incubation period; and for up to five days after symptom onset, to identify instances of using public transport whilst symptomatic, potentially exposing others. Data on demography and habitual use of buses and trams were also collected.

### Control subjects

After a patient with ARI was identified and enrolled as a case, the treating physician was asked to identify the next presenting patient (in chronological order) with an acute but non-respiratory condition who agreed to take part as the as a control. Controls completed a similar questionnaire, which asked about use of buses or trams in a comparator period, five days immediately prior to their visit to the doctor.

### Potential confounders

The following risk factors were evaluated as potential confounders: age [categorised as (0-24 yrs), (25-45 yrs), (46-65 yrs), (>65 years) to avoid creating age bands with missing data], which potentially influences immune status; gender; number of children in the household (all suggested to be associated with the risk of ARI); socioeconomic status, based on the 2007 Indices of Multiple Deprivation for each individual's area of residence (median electoral enumeration area-level IMD scores within each individual's sub-postcode of residence) [6]; self-reported co-morbidities (diabetes, cardiovascular disease, chronic lung disease, chronic renal disease); smoking status (current and ex-smokers for <10 years vs. stopped smoking >10 yrs and never-smokers); and influenza vaccination status for winter 2008/09.

### Power calculation

To attain statistical power of 80 percent at 5% significance, it was estimated that 184 study participants would be needed (92 cases and 92 controls) to detect an odds ratio of 2.5, based on previous estimates of the size of association [3]. The calculations assumed that about 21 percent of Nottingham's population use public transport (buses or trams) based on Department for Transport data [7].

### Ethical approval and consent

Ethical approval for this study was obtained from Leicestershire, Northamptonshire and Rutland Research Ethics Committee. All participants gave verbal consent before enrolment and were provided with written information.

### Statistical analysis

A univariable logistic regression analysis was carried out to investigate the association between potential risk factors and consultation for ARI. Multivariable analysis using a multiple logistic regression model was used to assess the strength of association between bus or tram use in the five days prior to symptom onset (cases) or consultation (controls) [binary categorical variable (yes/no)]. We also examined the relation between habitual frequency of public transport use (<once/week, 1-3 times/week, and >3 times/week) and ARI consultation. Frequency of public transport use was also modelled separately as a continuous variable to assess trends. Results have been expressed as odds ratios (OR) with 95% confidence intervals (CI). Age, gender and co-morbidity were included as *a priori *confounders (Model 1). Other variables included in the multivariable analysis (Model 2) were those that were statistically significant (p < 0.05) risk factors for ARI in univariable logistic regression analyses, or which modified the unadjusted OR for the main exposure variable (recent use of public transport) by at least ± 10% in a bivariate model (bivariate data available on request). Individuals providing incomplete responses were excluded from the analyses. We also tested for interactions between frequency of habitual public transport use, and recent use of public transport on the risk of ARI using the likelihood ratio test. All analyses were carried out using Stata^® ^10 (StataCorp, College Station, TX, USA).

Although we chose a period when community influenza activity had risen above baseline [5], our case definition was clinically based and it is highly likely that our cases of ARI included influenza and a range of other respiratory viruses. We chose a putative incubation period of five days and accordingly recorded public transport use in the five days before symptom onset as the likely window in which cases might have acquired their current infection from a fellow traveller, and a matching period prior to GP consultation in controls with non-respiratory conditions. We chose controls with other non-respiratory acute conditions because, if most controls were consulting with chronic conditions, this might have biased sampling towards patients less able or less willing to use public transport.

## Results and Discussion

During the study period (Dec 2^nd ^2008 - 15^th ^Jan 2009) we identified and obtained information by questionnaire from 72 patients with ARI and 66 controls with other acute non-respiratory conditions.

The median age of the sample population was 49 years (interquartile range: 24). Table 1 summarises the characteristics of cases and controls. Cases were more likely to be older, males, living with children and to have higher co-morbidity. Cases also appeared to be less frequent users of public transport use but this effect was not statistically significant when frequency of habitual public transport use was modelled as a continuous variable (p-trend = 0.845).

**Table 1:** Characteristics of cases and controls (n = 138)

Characteristic	Cases (%) (n = 72)	Controls (%) (n = 66)	Unadjusted OR (95% CI)
Age:			
1-24 yrs	11 (15)	4 (6)	1.00
25-45 yrs	32 (44)	16 (24)	0.73 (0.20-2.65)
46-65 yrs	25 (35)	32 (49)	0.28 (0.10-1.00)
>65 yrs	4 (6)	11 (17)	**0.13 (0.03-0.70)**
Missing	0 (0)	3 (4)	- *p-trend <0.001*
			
Gender:			
Male	37 (51)	18 (27)	1.00
Female	35 (49)	48 (73)	**0.35 (0.17-0.72)**
			
Child cohabitant:			
No	40 (56)	54 (82)	1.00
Yes	31 (43)	12 (18)	**3.48 (1.60-7.60)**
Missing	1 (1)	0 (0)	-
			
IMD score:			
Min-14 (least deprived)	6 (8)	2 (3)	1.00
15-29	18 (25)	15 (23)	0.40 (0.07-2.28)
30-44	36 (50)	26 (40)	0.46 (0.09-2.47)
45-max (most deprived)	8 (11)	14 (21)	0.19 (0.03-1.18)
Missing	4 (6)	9 (14)	- ***p-trend = 0.041***
			
Flu Vaccine 08/09:			
No	55 (76)	34 (52)	1.00
Yes	17 (24)	30 (45)	0.35 (0.17-0.73)
Missing	0 (0)	2 (3)	-
			
Smoking status:			
Non-smokers	38 (58)	47 (65)	1.00
Current smokers	28 (42)	25 (35)	0.72 (0.36-1.44)
			
Co-morbidity:			
No	56 (78)	41 (62)	1.00
Yes	16 (22)	25 (38)	**0.47 (0.22-0.99)**
			
Frequency of habitual bus/tram use:			
<once a week	40 (56)	24 (36)	1.00
1-3 times a week	9 (13)	22 (33)	**0.27 (0.11-0.69)**
>3 times a week	18 (25)	18 (27)	0.68 (0.29-1.57)
Missing	5 (7)	2 (3)	- *p-trend = 0.196*
			
Use of bus/tram in previous 5 days:			
No	34 (47)	31 (47)	1.00
Yes	38 (53)	31 (47)	1.10 (0.55-2.21)
Missing	0 (0)	4 (6)	-

Statistically significant results in bold

Table 2 presents the crude and adjusted odds ratios for the association between public transport use and acquisition of ARI. The univariate analysis showed that cases were 10 percent more likely to have travelled by public transport in the five days before symptom onset than controls (unadjusted OR: 1.10, 95% CI: 0.55-2.21). This association was not statistically significant. After adjusting for age, gender and co-morbidity (Model 1), cases were still 10 percent more likely than controls to have travelled by public transport in the five days before symptoms, but once again, this association was not statistically significant (adjusted OR: 1.09, 95% CI: 0.50-2.38).

**Table 2:** Odds ratios for the association between bus/tram use and ARI (n = 127)

Exposure	Cases (n = 67)	Controls (n = 60)	Unadjusted OR (95% CI)	**Model 1**^**a**^**: Adjusted OR (95% CI)**	Model 2: Adjusted OR (95% CI)
Bus/tram usage in previous 5 days:					
No	33 (49%)	31 (52%)	1.00	1.00	1.00
Yes	34 (51%)	29 (48%)	1.10 (0.55-2.21)	1.09 (0.50-2.38)	5.94 (1.33-26.5)^b^
					
Frequency of public transport use:					
<once a week	40 (60%)	24 (40%)	1.00	1.00	1.00
1-3 times a week	9 (13%)	20 (33%)	**0.27 (0.11-0.69)**	**0.27 (0.10-0.74)**	0.54 (0.15-1.95)^c^
>3 times a week	18 (27%)	16 (27%)	0.68 (0.29-1.57)	0.81 (0.32-2.08)	0.37 (0.13-1.06)^c^
			*p-trend = 0.196*	*p-trend = 0.463*	*p-trend = 0.057*

*Missing data have been excluded from the analysis; statistically significant results in bold; *^*a *^*adjusted for age, gender and co-morbidity; *^*b*^*adjusted for age, gender, co-morbidity, deprivation, child cohabitation, flu vaccination and frequency of habitual public transport use; *^c^*adjusted for age, gender, co-morbidity, child cohabitation, flu vaccination and deprivation*

Model 2 also adjusted for deprivation, child cohabitants, flu vaccination and habitual public transport use in addition to *a priori *confounders; this time the strength of the association increased, with cases nearly six times more likely to have travelled by public transport in the five days before symptoms; this was statistically significant (adjusted OR: 5.94, 95% CI: 1.33-26.47). After taking into account age, gender, co-morbidity, child cohabitants, flu vaccination and deprivation, there was still no statistically significant association between frequency of habitual public transport use and risk of consultation for ARI (p-trend = 0.057). Nevertheless, when examining frequency of habitual public transport use as a categorical variable, there was a trend towards a decreased risk of ARI consultation in persons who were more frequent habitual users (1-3 uses/week: adjusted OR = 0.54 95% CI: 0.15-1.95; >3 uses/week: adjusted OR = 0.37 95% CI: 0.13-1.06). The results of a separate logistic regression to explore the association between frequency of habitual public transport use and recent use are presented in Additional file 1; frequent habitual users were more likely to have been recent users but inclusion of habitual use in Model 2 nevertheless improved the overall fit compared with Model 1. We observed a significant interaction between regularity of public transport use and recent use on the risk of ARI (p = 0.019). We were unable to perform a complete stratified analysis by frequency of public transport use to explore this interaction further, because of the small sample size. However we present a limited stratified analysis in Additional file 2, which is consistent with our key finding that infrequent habitual use of public transport poses the greatest risk of ARI.

One of the most controversial issues in pandemic preparedness is the potential benefit conferred by public health measures, including social distancing strategies such as the closure of mass transportation systems [8,9]. No clear consensus has yet been reached about whether mass transportation systems should be closed during a pandemic; and the mildness of the influenza A/H1N1 pandemic in 2009 (which did not trigger transport closures) offers little prospect of shedding further light on this issue. When considering the possible policy impact, there is often confusion between the potential public health effects of closing urban transportation during a pandemic (thereby delaying population spread), versus the individual benefit of avoiding public transport (reduction in individual risk of infection). Much will also depend upon whether individuals will change their behaviour during a severe pandemic, by avoiding using public transport whilst symptomatic.

In this study we set out to explore the association between recent public transport use and the likelihood of acquiring acute respiratory infection (ARI) at an individual level. We found a significant association between ARI consultation and recent use of public transport; however, because of the small study size, the confidence intervals are wide and the magnitude of risk is not clearly defined. Furthermore, we detected a trend suggesting that the absolute risk of public transport in relation to ARI may be modified by frequency of habitual use, the risk being decreased among the most regular users. However the study lacked statistical power to explore this fully, using multiplicative modelling terms.

Our findings may suggest that whilst use of public transport in the winter potentially exposes travellers to respiratory viruses and increases the risk of ARI, the risk is offset among regular users who either acquire immunity against a range of respiratory viruses (or partial immunity sufficient to produce asymptomatic infections), presumably because they are more frequently exposed; or because they develop compensatory behaviours that reduce risk. An alternative methodological hypothesis would be that habitual users of public transport are exposed to respiratory viruses earlier in the winter season and so may have acquired protective immunity by the time this study began in early December.

If true, the implication of these findings for the 'normal' winter respiratory virus season is that occasional users of public have the greatest risk of acquiring ARI. The implications for pandemic preparedness may be somewhat different. Given a novel virus, against which there is lower background population immunity, the attenuating effect of regular public transport use would be diminished and the individual risk might be driven more by recent use of public transport and less by habitual patterns of usage. Thus the same protective effect of regular bus and tram use may not be present.

This study has a number of strengths and limitations. We used GP consultations for ARI as a proxy for 'all ARI' and acknowledge that because we selected only those ARIs, which necessitated medical attention (or selecting patients more likely to consult a GP with ARI), the findings should be interpreted carefully. Nevertheless, experienced primary care physicians identified all cases of ARI; their symptoms were cross-checked by the questionnaire tool and so misclassification bias seems highly unlikely. However without virological confirmation it is not possible to describe the range of underlying respiratory virus aetiologies in the cases in the study; thus the findings, while generalisable, are not specific to influenza. Controls were selected as the next patient with an acute non-respiratory condition, as determined by the chronological order of appointments in the practice. This may have introduced some bias if persons were seen in order of severity rather than by booking order. It is possible that if control patients with a non-respiratory complaint (e.g. worsening cardiac failure) in fact had underlying respiratory virus aetiology, this might have under-estimated the risk of public transport use. This study was performed in Nottingham where in general terms public transport use is more closely related to affluence than is the case in larger cities (e.g. London) with more extensive urban mass transportation networks. We also lacked data on subjects' normal working environments (e.g. lone working or in close contact with many people). However we adjusted for deprivation score in the multivariate analysis, which should account, to some extent, for affluence and job type. Whilst we obtained data on frequency of habitual public transport use, we did not obtain data on time of use (e.g. rush hour vs. off-peak) and passenger density may affect the likelihood of exposure to an infectious traveller. The study was somewhat underpowered due to the time and resource restrictions imposed by an undergraduate project. As participants were not asked whether they had acquired ARI symptoms earlier in the flu season, post-infection immunity in controls manifesting as sub-clinical ARI was not investigated. We were unable to address asymptomatic infection in the current study.

There are few published data available, against which this study can be meaningfully compared. Studies of transmission on board aircraft are not comparable because of differences in the number of 'passenger exchanges' per hour (very low compared with buses and trams), duration of journey, and ventilation [2]. The study by Kar-Purthayaska et al. concludes that transmission of pandemic influenza did not occur on board a school coach. However, these data are not strictly comparable because the population was less heterogeneous than on public transport, the two school groups may have remained separated, 'passenger exchange' was far lower and the outbreak occurred in summer, albeit with a journey time of 60 minutes [4]. In addition pandemic influenza A/H1N1 has been associated with a very high rate of asymptomatic infection in children [10]; so many may already have been immune or may have acquired asymptomatic infection following exposure. The most relevant comparator study, by Williams and colleagues, assessed the influence of regular public transport use on the risk of serologically confirmed influenza in hospital healthcare workers, finding a risk ratio of 1.06 (95% CI 0.69-1.63) in the 2006/07 winter season [3]. These investigators did not however report any findings about the frequency of habitual public transport use and did not investigate the influence of public transport use in the period immediately preceding illness onset. Our findings add new detail in relation to the possible role of public transport in relation to the acquisition of infection during a relevant incubation period and suggest that the frequency of habitual public transport use ought to be explored further in future studies.

Although causality can never be firmly proven on the basis of a small observational study, the effects we observed are biologically plausible including the diminution of risk with increasing habitual travel frequency. Buses and trams are generally poorly ventilated carriages of people sitting and standing in close proximity (particularly during peak travel periods) who, if travelling whilst symptomatic and exhibiting poor hand hygiene and cough etiquette, could spread respiratory virus infections via direct or indirect contact. Frequent public transport users regularly exposed to respiratory pathogens may be more likely to develop protective immunity. However, there still seems to be a risk associated with bus or tram use related to the period immediately preceding symptom onset. During a pandemic, when a novel respiratory virus is in circulation, the protective effect of habitual regular bus or tram use may be much reduced. Although small and limited in scope, this study nevertheless suggests that a larger more detailed investigation would be appropriate.

## Conclusions

The findings of this study suggest that recent use of public buses and trams is a significant individual risk factor for the acquisition of ARI (leading to GP consultation) in winter; this risk may be altered by frequency of habitual use but this requires further evaluation. The findings support current public advice to exercise good respiratory hygiene and existing pandemic guidance to refrain from making unnecessary journeys by public transport when symptomatic. The findings do not support the effectiveness of suspending mass urban transport systems as a pandemic countermeasure aimed at reducing or slowing population spread because, whatever the relevance of public transport is to individual-level risk, household exposure most likely poses a greater threat [3].

## List of Abbreviations

ARI: Acute respiratory infection; CI: Confidence interval; GP: General practitioner (primary care physician); IMD: Indices of multiple deprivation; OR: Odds ratio

## Competing interests

JSN-V-T holds an honorary consultant contract with the Funder and is a member of the UK Scientific Advisory Group for Emergencies (SAGE).

## Authors' contributions

JSN-V-T, JEE, ACH, JG and JT conceived and designed the study. JT, JEE and AH designed the questionnaire. JT, CJP, SK and AH executed the study. JT, JG, AH and PRM analysed the data. JT, PRM, JEE, and JSN-V-T wrote the manuscript; all other authors contributed to the manuscript and have read and approved the final version. JSN-V-T and PRM act as guarantors of the data.

## Authors Information

Joy Troko is an undergraduate medical student at the University of Nottingham, and contributed to this work as part of an intercalated BMedSci degree.

## Pre-publication history

The pre-publication history for this paper can be accessed here:

http://www.biomedcentral.com/1471-2334/11/16/prepub

## Supplementary Material

Additional file 1 **Relationship between frequency of habitual public transport use and recent use**. The results of a separate logistic regression to explore the association between frequency of habitual public transport use and recent use (n = 131). Click here for file

Additional file 2 **Association between recent bus/tram use and ARI stratified by habitual use**. Limited stratified analysis showing the association between recent bus/tram use and ARI stratified by habitual use (n = 127). Click here for file
