# Supplementary material for: Is public transport a risk factor for acute respiratory infection?
Source: BMC Infect Dis. 2011 Jan 14;11:16. doi: 10.1186/1471-2334-11-16 (PMC3030548; doi:10.1186/1471-2334-11-16)
Supplement: Additional file 1 — Relationship between frequency of habitual public transport use and recent use. The results of a separate logistic regression to explore the association between frequency of habitual public transport use and recent use (n = 131). [file 1471-2334-11-16-S1.DOCX]

**Additional file 1: Relationship between frequency of habitual public transport use and recent use**

| Frequency of public transport use | Unadjusted OR (likelihood of recent travel by public transport compared to infrequent users of public transport (<1/week) | Lower Limit 95% CI | Upper Limit 95% CI |
| --- | --- | --- | --- |
| 1-3 times a week | 22.5 | 7.4 | 68.8 |
| >3 times a week | 43.2 | 12.5 | 149.2 |

**Logistic regression model: n=131**
