# Supplementary material for: Is public transport a risk factor for acute respiratory infection?
Source: BMC Infect Dis. 2011 Jan 14;11:16. doi: 10.1186/1471-2334-11-16 (PMC3030548; doi:10.1186/1471-2334-11-16)
Supplement: Additional file 2 — Association between recent bus/tram use and ARI stratified by habitual use. Limited stratified analysis showing the association between recent bus/tram use and ARI stratified by habitual use (n = 127). [file 1471-2334-11-16-S2.DOCX]

**Additional file 2: Relationship between recent bus/tram use and ARI stratified by habitual use**

| **Stratified by habitual public transport use** | **Unadjusted OR**  **(95% CI)** | **Model 1^a^:**  **Adjusted OR (95% CI)** | **Model 2^b^:**  **Adjusted OR**  **(95% CI)** |
| --- | --- | --- | --- |
| < once a week  1-3 times a week  >3 times a week | 2.75 (0.53-14.20)  0.80 (0.42-1.52)  0.79 (0.49-1.29) | 2.73 (0.44-17.02)  0.72 (0.14-3.63)  0.77 (0.47-1.29) | 22.25 (0.81-612.14)  - ^c^  0.89 (0.21-3.81) |

*Logistic regression model: n=127*

*Missing data have been excluded from the analysis; statistically significant results in bold; ^a^ adjusted for age, gender and co-morbidity; ^b^ adjusted for age, gender, co-morbidity, deprivation, child cohabitation and flu vaccination; ^c^ could not be calculated because of insufficient data*
